# External ambient radiation exposure and cancer mortality trends around Qinshan nuclear power plant phase I: long-term study before and after service extension

**DOI:** 10.3389/fpubh.2026.1780605

**Published:** 2026-05-08

**Authors:** Yihang Li, Yiyao Cao, Xiaojing Zhang, Jiadi Guo, Junjie Xu, Shunfei Yu, Hua Zou, An Zhang, Hong Ren

**Affiliations:** 1 Zhejiang Provincial Center for Disease Control and Prevention, Hangzhou, Zhejiang, China; 2 School of Public Health, Suzhou Medical College, Soochow University, Suzhou, China; 3 Shandong Nuclear and Radiation Safety Monitoring Center, Jinan Shandong, China; 4 Haiyan County Center for Disease Control and Prevention, Haiyan, Zhejiang, China; 5 School of Public Health, Hangzhou Medical College, Hangzhou, China

**Keywords:** ambient radiation, cancer mortality, long-term environmental monitoring, Qinshan nuclear power plant, radiation exposure assessment

## Abstract

This study evaluates the ambient external radiation levels and their association with public health around the Qinshan Nuclear Power Plant (QNPP) before and after the extended service of its Phase I project. By monitoring the cumulative ambient external exposure around QNPP from 2010 to 2023 and analyzing the health data of residents in Haiyan County from 2012 to 2022, changes in cancer mortality-related rates were examined. Results showed that during the study period, ambient radiation around QNPP remained at background levels. Although the annual cumulative ambient dose increased after the extended service, the annual effective dose and excess risk still remained well below the standard limits. From 2012 to 2022, the crude cancer mortality rate and age-standardized mortality rate among residents living near QNPP were slightly higher than the average levels in Zhejiang Province, with males having higher rates than females. However, an overall non-statistically significant downward temporal trend was observed (ASMRC: APC=−7.0, 95% CI: −14.6–1.3, *p* > 0.05). The mortality rates of leukemia and thyroid cancer also showed non-statistically significant decreasing trends over time. In short, given the constraints of this ecological and descriptive study, no statistically significant association was observed between the operation of QNPP (including its pre- and post-service extension phases) and either environmental radiation anomalies or public health risks in the surrounding area. Nevertheless, continuous long-term monitoring remains essential to safeguard environmental quality and public health.

## 1 Introduction

The 2015 Paris Agreement requires parties to formulate NDC emission reduction targets, limiting global warming to within 2 °C compared to pre-industrial levels and achieving near-zero carbon electricity generation by the middle of this century. As a dispatchable low-carbon energy source, nuclear power plays a crucial role in this transition (1). As a party to the Agreement, China is actively advancing its “dual carbon” goals through the development of non-fossil energy sources, including nuclear power (2). Currently, there are 56 operational nuclear power units in China (excluding the Taiwan region), with a total installed capacity of 58,218.34 MWe (rated installed capacity) (3).

However, numerous epidemiological studies (4–12) have indicated that workers and residents living near nuclear power plants face increased health risks even when exposed to radiation levels within regulated dose limits. Moreover, following the Fukushima nuclear accident in Japan, people worldwide have become more concerned about such potential risks (13) and expressed varying degrees of anxiety. For this reason, many countries have implemented radioactive monitoring and radiation risk assessment programs before and after the operation of nuclear power plants (14, 15).

Located in Haiyan County, Zhejiang Province, China, Qinshan Nuclear Power Plant (QNPP) is the country's first independently designed and constructed 300 MW pressurized water reactor nuclear power plant. It was commissioned in 1991 with a 30-year design life and approved for a 20-year extension in 2021, allowing safe operation through 2041 (16). While offering high efficiency and low carbon benefits, its operation may involve radioactive releases (17). Hence, tracking radiation exposure and radioactivity levels during this period is critical—it enables timely understanding of radioactive conditions, accurate trend identification, and thus supports timely understanding of radiological conditions and helps protect public health and environmental quality.

Public exposure around nuclear power plants encompasses both external and internal radiation pathways (18). External radiation exposure is mainly caused by radioactive sources in the surrounding environment (19), including artificial radioactive emissions, plumes and fallout, which continuously emit radiation into the environment and potentially alter local ambient radiation levels. Internal exposure, by contrast, is often driven by specific radionuclides—such as tritium—via ingestion of contaminated food or water, or inhalation of radioactive substances. According to the Bergonie-Tribondeau Law, cell sensitivity to radiation varies with cellular proliferation rate, differentiation status, and individual biological characteristics. These factors contribute to a broad spectrum of potential health effects that extend beyond cancer, including reproductive outcomes and other radiation-sensitive endpoints.

Given the presence of nuclear power units in Haiyan County, the region is susceptible to the combined impacts of multiple radiation pathways. However, comprehensive long-term data that integrates external and internal radiation exposure, individual radiation sensitivity, and multiple health outcomes around QNPP Phase I remains scarce across both pre- and post-extension periods. In the current study, we focus on assessing the health impacts of external radiation exposure in Haiyan County, while recognizing this as a foundational step toward more holistic future investigations that incorporate internal exposure pathways and a broader range of health endpoints. To this end, this study aims to provide data on cumulative external ambient radiation doses around the Phase I project of QNPP before (2010–2021) and after (2022–2023) its extended service. Meanwhile, using de-identified authorized public health surveillance data (without involving individual private information or clinical decision interference), it analyzes the cancer mortality rates of residents in Haiyan County for the period of 2012–2022, especially the mortality rates of radiation-sensitive cancers. Based on the monitoring results of cumulative external ambient radiation doses, it evaluates the annual effective dose (*AED*_e_) and excess risk (*ER*_e_), and analyzes the cancer mortality rates of the residents living near QNPP. The results of this study can serve as basic data for assessing the impacts of future operation of QNPP on human health risks and the surrounding environment.

## 2 Methods and materials

### 2.1 Sampling and analysis

The external ambient radiation monitored in this study primarily comprises gamma and scattered beta radiation. Thermoluminescent dosimeters (TLDs) were specifically calibrated to quantify these radiation types but do not capture internal exposure or measure specific radionuclides. Accordingly, this study focused on external ambient radiation exposure by quantifying cumulative ambient dose (*CAD*) using TLDs. Internal exposure pathways (e.g., ingestion or inhalation of radionuclides via food, water, air) were not assessed, as they require targeted sampling (e.g., food, soil, biological specimens) and analytical methods beyond the scope of this long-term environmental monitoring program. The study was conducted from January 2010 to December 2023, divided into two phases: the pre-expansion phase (2010–2021) and the post-expansion phase (2022–2023) of QNPP Phase I. TLDs were deployed on the 1st −5th days of the 1 month of each quarter and retrieved on the 25th −30th days of the last month of that quarter to ensure cycle consistency, with quarterly monitoring aligned with natural quarters.

**Figure 1:**
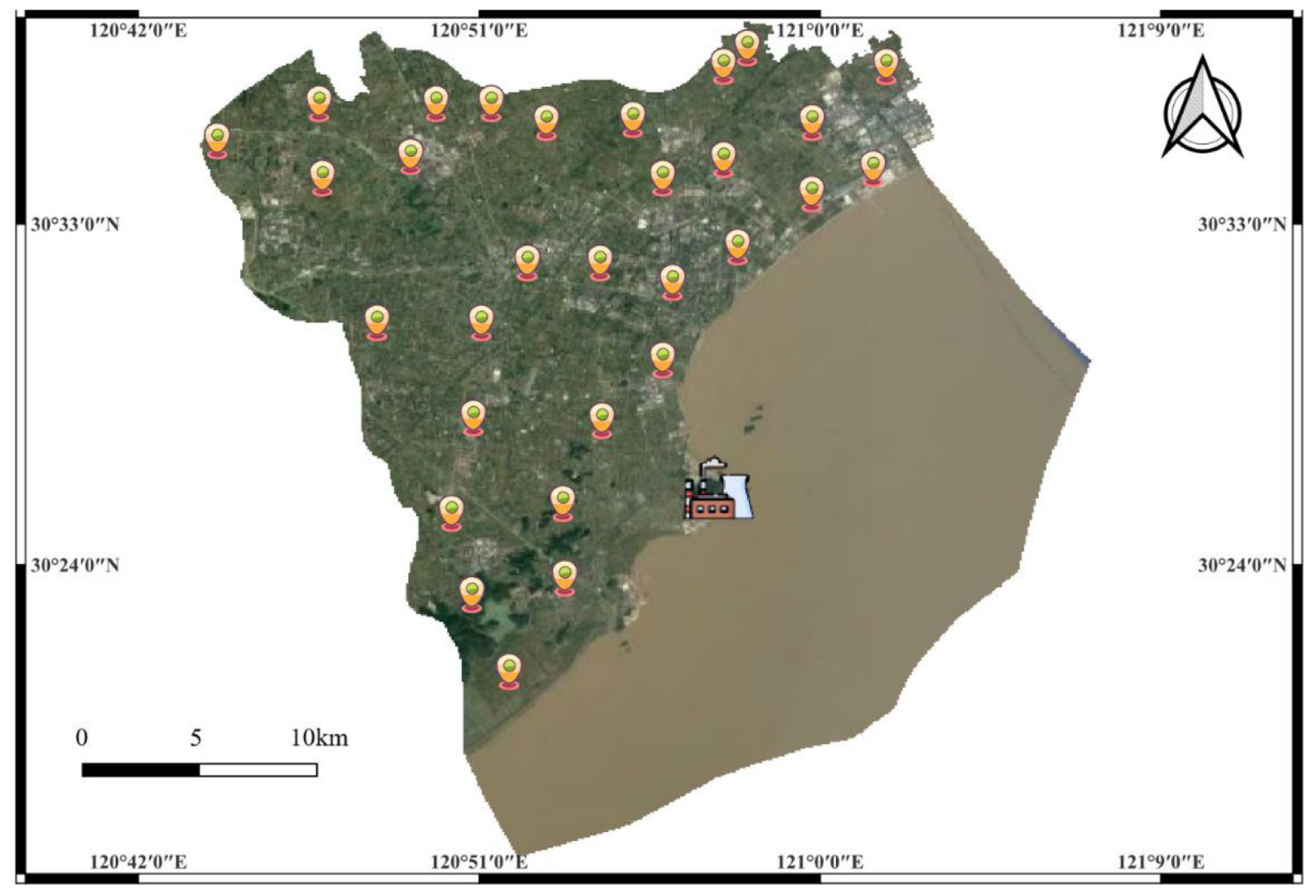
Schematic diagram of the distribution of monitoring stations around the phase I project of Qinshan nuclear power plant.

The sampling was centered on QNPP Phase I, with 30 monitoring stations established across three concentric circular zones (0–10 km, 10–20 km, and 20–30 km), and 10 stations in each zone, stations were spaced at least 2 km apart to avoid spatial correlation, with dense layout in the 0–10 km zone and terrain-representative distribution in 10–30 km (see Figure 1). At each station, two TLDs (LiF: Mg, Cu, P type) were deployed simultaneously 2 m above ground on tree trunks, facing open sky, avoiding dense canopy and nearby shielding structures to minimize vegetation and obstacle effects. The zoning design was guided by three considerations: (1) Ten dense monitoring stations were set up in the 0–10 km direct influence zone; (2) Monitoring stations in the 10–30 km indirect influence zone covered diverse terrain types to ensure representativeness; (3) The distance between adjacent monitoring stations was generally no less than 2 kilometers to avoid overlap, with exceptions in areas requiring denser deployment to enhance monitoring accuracy. This design complied with the ICRP Publication 103 (2007) (20) and the GB 6249-2011 standard (21). At the end of each quarterly cycle, all TLDs were retrieved, measured, and their data were recorded.

### 2.2 Quality assurance and quality control

To ensure the accuracy and reliability of the monitoring data, this study implemented strict quality assurance and quality control measures. Before each deployment, a Type C TLD precision annealing furnace was used to anneal the TLDs to eliminate residual doses, and the measurement process strictly followed standard operating procedures. During the measurement of TLDs, the operating protocol for the dosimeter (RGD 3B), manufactured by the Institute of Chemical Defense, Academy of Military Sciences, Chinese People's Liberation Army (Beijing, China), was strictly followed, including preheating stabilization, background correction, and signal amplification calibration. The entire detection system (including TLDs and reading instruments) is calibrated annually by the Zhejiang Institute of Metrology, and all performance indicators meet the requirements of the national standard GB/T 10264-2014 (20). Meanwhile, the team participates in the national blind sample comparison for external exposure dose organized by the National Institute for Radiological Protection, Chinese Center for Disease Control and Prevention, and has repeatedly achieved excellent results.

### 2.3 Dose assessment

Annual effective dose (*AED*, mSv/y) is a radiation protection quantity recognized as a useful tool for assessing risks of radiation exposure and formulating radioactive contamination control policies. In this study, the annual effective dose caused by external ambient exposure (*AED*_e_, mSv/y) was used to quantify the cumulative dose in the environment surrounding the Phase I Project of the QNPP. Age-related *AED*_e_ and Excess risk (*ER*_e_) were calculated using Equations (1) and (2) to evaluate risks for radiation-exposed populations.


$$\begin{array}{l} AED_{e}=CAD\;\times \;O \end{array}$$ (1)

In the Equation (1), CAD represents the cumulative ambient dose (mSv); O denotes the age-related outdoor occupancy factor derived from early studies of the Chinese population (21, 22). This dataset covers 31 provinces and cities in China with a sample size exceeding 100,000, which is consistent with the activity characteristics of residents in Haiyan County, Zhejiang Province, justifying its application in this study. The values of O for different age groups are shown in Table 1.

**Table 1:** The age-dependent outdoor occupancy factors.

Age (y)	1–2	2–3	3–4	4–5	5–6	6–9	9–12	12–15	15–18	≥18
O	0.115	0.131	0.122	0.100	0.100	0.069	0.059	0.064	0.064	0.133

Excess risk (*ER*_e_) refers to the excess incidence rate of specific health effects associated with radiation exposure, estimated using Equation (2).


$$\begin{array}{l} ER_{e}=AED\;\times \;RF\;\times \;DL \end{array}$$ (2)

Among them, *RF* is the hazard-adjusted nominal risk coefficient for cancer and genetic effects following low-dose-rate radiation exposure. The ICRP Report 103 recommends a cancer risk coefficient of 5.5 × 10^−5^/mSv and a genetic effect risk coefficient of 0.2 × 10^−5^/mSv (23), which is in line with international general standards and ensures the authority of dose conversion. *DL* is the lifespan (70 years). Notably, this model has been validated to demonstrate strong applicability in low-dose radiation risk assessment for nuclear power plants, with an R^2^ value typically exceeding 0.85 and a prediction error of less than 10% (17, 19). Furthermore, the *O* in this study has further enhanced the model's suitability for the research area. It is important to acknowledge, however, that both the *O* and *RF* are population-averaged metrics. They do not account for individual variability in radiation sensitivity as described by the Bergonie-Tribondeau Law, which may introduce uncertainties in individual-level risk estimates—this limitation is further discussed in the Discussion section.

The above approach (Equation (2)) has shown strong applicability in low-dose radiation risk assessment for nuclear power plants, with an R^2^ value typically exceeding 0.85 and a prediction error of less than 10% (17, 19). It is important to acknowledge, however, that both *O* and *RF* are population-averaged metrics. They do not incorporate individual variability in radiation sensitivity as described by the Bergonie-Tribondeau Law, which may introduce uncertainties in individual-level risk estimates. This limitation is further addressed in the Discussion section.

### 2.4 Analysis of cancer mortality

Health data of residents in Haiyan County from 2012 to 2022 were obtained from the Zhejiang Provincial Chronic Disease Management System. All diagnosed cancer cases were coded according to the 10^th^ edition of the International Classification of Diseases (ICD-10). Mortality rates for all combined cancer sites (ICD-10: C00-C97), leukemia (ICD-10: C91-95), and thyroid cancer (ICD-10: C73) were analyzed. Leukemia and thyroid cancer were selected for focused analysis due to their radiosensitivity (24). Descriptive statistical analyses were performed on overall mortality from malignant tumors and mortality from radiosensitive cancers.

The model (Version 5.2.0.0) was used to analyze temporal trends in cancer mortality. A maximum of 1 joinpoint was allowed, and the final model was selected using permutation tests and the Akaike Information Criterion (AIC). No significant joinpoints were identified during 2012–2022; thus, a single annual percentage change (APC) was calculated to represent the overall annual trend. APC >0 indicates an upward trend and APC < 0 indicates a downward trend. To enable comparative analyses across different age structures, Chinese age-standardized mortality rates for cancer (ASMRC) and World age-standardized mortality rates (ASMRW) were calculated based on the 2,000 Chinese standard population and the WHO 2000 standard population, respectively.

### 2.5 Ethical statement

This work is an extension of the original research project for which a medical ethics review form has been provided. As a retrospective analysis using de-identified authorized public health surveillance data (without involving individual private information or clinical decision interference), the ethical approval granted for the original project (issued by the Zhejiang Provincial Center for Disease Control and Prevention) is fully applicable. The Zhejiang Provincial Center for Disease Control and Prevention has confirmed that the exemption of ethical review and informed consent for the original project also extends to this study. All research procedures were conducted in strict compliance with the ethical guidelines of the Declaration of Helsinki.

## 3 Results

### 3.1 Cumulative ambient dose

Table 2 presents the *CAD* values for the first-phase project of QNPP before (2010–2021) and after (2022–2023) its service life extension. The data from 2012 to 2020 were directly obtained from previously published studied conducted by our research team (18). During 2010–2021, the mean quarterly radiation dose rates were 0.082, 0.083, 0.110, and 0.075 mSv, respectively, with an annual total mean of 0.350 mSv, showing only minor fluctuations throughout the period. For 2022–2023, the mean quarterly values were 0.229, 0.072, 0.172, and 0.203 mSv, respectively, with an annual total mean of 0.675 mSv. Except for the second quarter, the mean values of the remaining quarters were slightly higher than those of the corresponding quarters in 2010–2021. Notably, *CAD* values during 2022–2023 were moderately higher than in previous years. This elevation mainly caused by large-scale construction near one monitoring station, along with minor station adjustments and meteorological variability. The distinct peak in 2018 coincided with higher annual precipitation in Haiyan County (25), a well-recognized factor that can temporarily increase *CAD*. All measured values remained far below the global average annual natural background radiation (approximately 3.0 mSv/a) (26) and national average for Chinese public (approximately 2.3 mSv/a) (27). Additionally, both periods were lower than the pre-operation annual *CAD* of 0.832 mSv at the QNPP (28). These findings indicate that external ambient radiation in the study area remained at background levels.

**Table 2:** *CAD* around QNPP and statistical analysis for the temporal trend from 2010 to 2023.

Year	***CAD*** **(mSv)**				Total
	First quarter	Second quarter	Third quarter	Fourth quarter	
2010	0.051	0.125	0.300	0.112	0.588
2011	0.078	0.072	0.046	0.071	0.267
2012	0.066	0.082	0.059	0.037	0.244
2013	0.105	0.073	0.066	0.066	0.310
2014	0.087	0.076	0.075	0.052	0.290
2015	0.073	0.044	0.12	0.056	0.290
2016	0.078	0.058	0.076	0.066	0.278
2017	0.059	0.008	0.104	0.093	0.264
2018	0.211	0.122	0.167	0.103	0.603
2019	0.059	0.061	0.067	0.124	0.311
2020	0.059	0.182	0.104	0.057	0.402
2021	0.065	0.091	0.133	0.067	0.356
Mean	0.082	0.083	0.110	0.075	0.350
2022	0.151	0.081	0.092	0.204	0.528
2023	0.306	0.063	0.251	0.202	0.822
Mean	0.229	0.072	0.172	0.203	0.675

### 3.2 Age-related annual effective dose (*AED*_*e*_) and excess risk (*ER*_*e*_)

To assess the potential impact of external ambient radiation in the study area on human health, we calculated and summarized age-related *AED*_e_ and *ER*_e_, as shown in Table 3. The results indicate that the average *AED*_e_ before the service extension of QNPP Phase I ranged from 20.7 × 10^−3^ mSv/y to 46.6 × 10^−3^ mSv/y; but slightly increased to 39.8 × 10^−3^ mSv/y to 89.8 × 10^−3^ mSv/y after the extension.

**Table 3:** Age—dependent *AED*_*e*_ and *ER*_*e*_ induced by environmental exposure of people around QNPP from 2010 to 2023.

Age(y)	***AED***_**e**_**(**×**10**^**−3**^**mSv/y)**						***ER***_**e**_**(**×**10**^**−5**^**)**					
	**2010–2021 min**	**2022–2023 min**	**2010–2021 max**	**2022–2023 max**	**2010–2021 avg**	**2022–2023 avg**	**2010–2021 min**	**2022–2023 min**	**2010–2021 max**	**2022–2023 max**	**2010–2021 avg**	**2022–2023 avg**
1–2	28.1	60.7	69.3	94.5	40.3	77.6	10.8	23.4	26.7	36.4	15.5	29.9
2–3	32.0	69.2	79.0	107.7	45.9	88.4	12.3	26.6	30.4	41.5	17.7	34.0
3–4	29.8	64.4	73.6	100.3	42.7	82.4	11.5	24.8	28.3	38.6	16.5	31.7
4–5	24.4	52.8	60.3	82.2	35.0	67.5	9.4	20.3	23.2	31.6	13.5	26.0
5–6	24.4	52.8	60.3	82.2	35.0	67.5	9.4	20.3	23.2	31.6	13.5	26.0
6–9	16.8	36.4	41.6	56.7	24.2	46.6	6.5	14.0	16.0	21.8	9.3	17.9
9–12	14.4	31.2	35.6	48.5	20.7	39.8	5.5	12.0	13.7	18.7	8.0	15.3
12–15	15.6	33.8	38.6	52.6	22.4	43.2	6.0	13.0	14.8	20.3	8.6	16.6
15–18	15.6	33.8	38.6	52.6	22.4	43.2	6.0	13.0	14.8	20.3	8.6	16.6
>18	32.5	70.2	80.2	109.3	46.6	89.8	12.5	27.0	30.9	42.1	17.9	34.6

Among children under 18 years' old, 2–3-year-old children had the highest average *AED*_e_ due to the longest outdoor activity time, and *AED*_e_ values thereafter decreased with age. The average *AED*_e_ values before and after the extended service of QNPP Phase I were both lower than the doses specified in Chinese national standards (0.25 mSv/y) (29) and the global average annual dose from the natural background radiation (approximately 2.4 mSv/y) (30). Additionally, the annual average *ER*_e_ values ranged from 8.0 × 10^−5^ to 17.9 × 10^−5^ before the extension and 15.3 × 10^−5^ to 34.6 × 10^−5^ after the extension, with the same age distribution pattern as *AED*_e_.

### 3.3 Cancer mortality rates around QNPP

#### 3.3.1 Mortality rate of all cancer sites combined

From 2012 to 2022, a total of 15,781 malignant tumor deaths were reported in the vicinity of QNPP, with a crude mortality rate of 377.38 per 100,000, an ASMRC (age-standardized mortality rate based on China's 2,000 standard population) of 196.11 per 100,000, and an ASMRW (age-standardized mortality rate based on WHO's 2,000 standard population) of 190.21 per 100,000. Of these, 8,357 deaths occurred in males (crude mortality rate: 405.95 per 100,000; ASMRC: 202.18 per 100,000; ASMRW: 199.96 per 100,000), and 7,424 in females (crude mortality rate: 349.68 per 100,000; ASMRC: 195.01 per 100,000; ASMRW: 185.85 per 100,000), indicating a higher mortality rate in males than in females. The crude mortality rate, ASMRC, and ASMRW of malignant tumors among residents around QNPP were slightly higher than those in Zhejiang provincial cancer registration areas from 2010 to 2014 (crude mortality rate: 186.06 per 100,000; ASMRC: 103.02 per 100,000; ASMRW: 101.73 per 100,000) (31).

This difference may be attributed to confounding factors such as regional socioeconomic status, access to medical resources, and dietary habits, rather than radiation exposure (32). As these provincial comparisons are descriptive and unadjusted for key confounders, they should be interpreted with caution. Notably, the overall mortality rate for all malignant tumors among residents living near QNPP exhibited a downward trend during the study period (2012–2022) (ASMRC: APC = −7.0, 95% CI: −14.6–1.3; ASMRW: APC = −6.8, 95% CI: −14.0–1.1). However, since the 95% CIs of the APCs all included 0, this downward trend is not statistically significant (*p* >0.05) and requires further verification through long-term monitoring. Wide 95% CIs were observed due to the limited study period and small case numbers for radiosensitive cancers, which reduced statistical power to detect small changes.

#### 3.3.2 . Mortality rate of radiosensitive cancer

As shown in Table 4, from 2012 to 2022, a total of 253 leukemia deaths were reported in Haiyan County, with a crude mortality rate of 6.05 per 100,000, an ASMRC of 5.35 per 100,000, and an ASMRW of 6.90 per 100,000 (Table 5). These values were slightly higher than the relevant data of Zhejiang Province in 2022 (crude mortality rate: 4.38 per 100,000; ASMRC: 3.15 per 100,000; ASMRW: 3.39 per 100,000) (33). Higher leukemia mortality in the local area may be related to genetic predisposition, chemical exposures, viral infections, and other non-radiation factors. During the same period, only 14 thyroid cancer deaths were reported in Haiyan County, with a crude mortality rate of 0.33 per 100,000, an ASMRC of 0.32 per 100,000, and an ASMRW of 0.25 per 100,000. These mortality rates were lower than the provincial level in Zhejiang (0.46–0.49 per 100,000) (32). Joinpoint regression analysis revealed that mortality rates for both cancers exhibited downward trends over the study period, however, these trends were not statistically significant. For example, in terms of ASMRC, leukemia: APC = −1.41%, 95% CI: −8.6–6.4, *p* = 0.72; thyroid cancer: APC = −6.12%, 95% CI: −23.0–14.3, *p* = 0.57. The lack of significance in the overall trends may be attributed to the small sample size and substantial annual fluctuations, particularly the occurrence of zero deaths in some years for thyroid cancer.

**Table 4:** Total mortality from all cancers for residents living near QNPP from 2012 to 2022. (1/100 000).

Year	All				Male				Female			
	Deaths	Crude rate	ASMRC	ASMRW	Deaths	Crude rate	ASMRC	ASMRW	Deaths	Crude rate	ASMRC	ASMRW
2012	1,290	343.90	197.86	190.11	722	388.90	225.49	219.42	568	299.80	178.79	169.44
2013	1,280	340.38	190.65	184.39	718	377.56	216.17	212.33	562	295.53	173.1	164.16
2014	1,567	415.21	229.83	222.22	820	439.94	238.27	236.07	747	391.07	227.69	215.24
2015	1,486	392.48	217.52	206.79	805	430.85	233.27	224.62	681	355.11	207.98	195.2
2016	1,575	414.90	219.36	211.65	808	431.70	224.38	220.43	767	398.56	219.31	208.18
2017	1,608	422.01	220.59	209.76	814	433.62	211.21	205.06	794	410.73	234.15	218.54
2018	1,642	429.91	228.16	215.77	822	437.35	220.61	211.77	820	422.70	239.61	224.09
2019	1,826	477.41	252.85	237.53	918	488.47	235.27	226.06	908	466.73	273.79	252.75
2020	1801	470.26	241.62	228.9	852	453.38	208.87	202.33	949	486.52	275.97	257.86
2021	773	201.79	68.98	68.62	504	268.57	97.71	96.98	269	137.66	45.22	46.03
2022	933	243.37	81.87	82.17	574	306.12	110.31	111.98	359	183.29	58.18	58.19
Total	15,781	377.38	196.11	190.21	8,357	405.95	202.18	199.96	7,424	349.68	195.01	185.85
APC (%)	–	−2.7	−7.0	−6.8	–	−2.1	−6.3	−6.2	–	−3.8	−8.5	−8.1
95 CI% (%)	–	−8.2 to 3.2	−14.6 to 1.3	−14.0 to 1.1	–	−5.7 to 1.7	−11.0 to 1.3	−10.7 to 1.4	–	−11.7 to 4.8	−19.1 to 3.5	−18.2 to 3.3

**Table 5:** The mortality rates of leukemia and thyroid cancer for residents living near QNPP from 2012 to 2022. (1/100,000).

Year	Leukemia				Thyroid cancer			
	Deaths	Crude rate	ASMRC	ASMRW	Deaths	Crude rate	ASMRC	ASMRW
2012	17	4.53	3.04	2.80	0	0	0	0
2013	20	5.32	4.59	5.32	1	0.27	0.40	0.27
2014	24	6.36	4.98	6.22	3	0.79	1.18	0.87
2015	22	5.81	4.42	4.15	0	0	0	0
2016	21	5.53	3.25	3.18	2	0.53	0.54	0.41
2017	23	6.04	3.90	4.60	2	0.52	0.78	0.59
2018	21	5.50	4.06	3.77	0	0	0	0
2019	29	7.58	4.98	5.08	1	0.26	0.21	0.18
2020	35	9.14	5.18	5.21	0	0	0	0
2021	17	4.44	1.51	1.56	3	0.78	0.25	0.19
2022	24	6.26	2.73	2.72	2	0.52	0.18	0.19
Total	253	6.05	5.35	6.90	14	0.33	0.32	0.25
APC (%)	–	0.92	−1.41	−1.13	–	−5.83	−6.12	−6.05
95 CI% (%)	–	−5.8 to 7.9	−8.6 to 6.4	−8.1 to 6.4	–	−22.7 to 13.9	−23 to 14.3	−22.9 to 14

## 4 Discussion

### 4.1 Contextualization with low-dose radiation epidemiology

Our results are consistent with major international studies on health effects of low-dose radiation. The UNSCEAR 2020 Report emphasizes that epidemiological evidence for radiation-induced cancer remains inconclusive at cumulative doses below 100 mSv, as the biological effects of low-dose ionizing radiation are not yet fully elucidated (34). Similarly, the 15-country collaborative study of nuclear industry workers (7) found no significant increase in cancer risk at dose levels comparable with the annual effective dose (*AED*_e_ ≤ 0.09 mSv/y) estimated in our study. These studies collectively support the interpretation that low-level external ambient radiation exposure around QNPP is unlikely to be associated with measurable changes in cancer mortality.

Notably, our findings are also consistent with long-term monitoring results from other nuclear power plants worldwide. For example, Hamlat et al. (19) reported that independent environmental monitoring around Canadian nuclear power plants showed no significant radiation-related health risks, with public doses well below regulatory limits. Similarly, Ren et al. (17) found no detectable association between operation of China's Sanmen Nuclear Power Plant and surrounding public health outcomes, further validating the generalizability of our conclusions.

### 4.2 Uncertainties in dose assessment

Several key parameters should be considered in the uncertainties of dose assessment: (1) TLD detectors were mounted on tree trunks at a height of 2 m. Potential bias from vegetation shielding, trunk heterogeneity, and local microclimate may introduce less than 10% variability in measured cumulative ambient dose. This magnitude of uncertainty is consistent with routine environmental radiation monitoring practice. (2) Age-related outdoor occupancy factors (*O*) were derived from national population surveys (21, 22), but local seasonal variations in outdoor activity (e.g., higher outdoor time in summer in Zhejiang) may introduce an estimated ± 10% error in *AED*_e_ calculations. (3) The 70-year lifespan (*DL*) assumption follows ICRP 103 recommendations (23), but it is conservative relative to the average life expectancy of Zhejiang residents (82.55 years in 2024) (35), leading to an approximately 15% underestimation of *ER*_e_. (4) The hazard-adjusted nominal risk coefficient (*RF*) is population-averaged (5.5 × 10^−5^/mSv for cancer, ICRP, 2007), without incorporating inter-individual variability in genetic susceptibility (e.g., variations in DNA repair genes) or lifestyle modifiers (e.g., smoking, alcohol consumption) that may alter radiation sensitivity. This limitation introduces uncertainty when extrapolating population-level coefficients to individual-level risk estimates. However, these uncertainties are within the acceptable range of low-dose radiation assessment (36) and do not alter the overall conclusion that environmental radiation around QNPP remained within background levels and well below regulatory limits.

### 4.3 Individual sensitivity

We acknowledge the Bergonie-Tribondeau Law, which highlights that cell radiation sensitivity varies with proliferation rate, differentiation status, and individual genetic background. This variability implies that population-level dose assessments may not reflect individual risk differences—for example, vulnerable groups such as pregnant individuals or those with genetic predispositions to radiation-related effects may exhibit distinct responses. However, due to the ecological design of this study, individual-level data could not be incorporated to quantify this variability.

### 4.4 Methodological limitations and interpretation caveats

#### 4.4.1 Methodological limitations

This study adopts an ecological and descriptive design, which is inherently observational and therefore unable to establish causal relationships between radiation exposure and cancer mortality. Accordingly, the conclusion of “no statistically significant association” reflects only the absence of detectable links under the current monitoring framework and data scope, rather than definitive evidence that no biological effect exists. This aligns with the inherent limitations of ecological studies, where population-level correlations cannot be extrapolated to individual-level causal effects (37).

In addition, the post-extension follow-up period (2022–2023) is far shorter than the latency of radiation-induced cancers, and radiation-induced solid tumors have a long latency period (23). We do not infer long-term health effects from post-extension radiation data; instead, these data serve as a continuation of baseline monitoring to track short-term radiation trends. The long-term safe operation record of QNPP, over 100 reactor-years without nuclear safety incidents (38), provides supplementary evidence consistent with no adverse radiological impacts during post-extension operation.

This study primarily focuses on cancer mortality. However, radiation exposure can influence a wider range of public health outcomes beyond this endpoint. Reproductive health effects—such as pregnancy loss and fetal development abnormalities—are particularly sensitive to low-dose radiation, consistent with the high proliferation rate of reproductive cells and aligned with the Bergonie-Tribondeau Law. Unfortunately, standardized reproductive health data were not available through the Zhejiang Provincial Chronic Disease Management System for the study period (2012–2022), which prevented the inclusion of these outcomes into our analysis. Moving forward, research should collaborate closely with maternal and child health institutions to gather prospective reproductive health data. Such partnership would enable a more comprehensive assessment of the broader health effects of low-dose radiation.

#### 4.4.2 Interpretation caveats

The present exposure assessment focuses on external ambient radiation measured using TLDs. Internal exposure pathways (e.g., ingestion or inhalation of radionuclides via food, water, air) were not included, as they require targeted sampling of food, soil, or biological specimens beyond the scope of this long-term environmental monitoring program. Consequently, conclusions based on the dose assessment in this study should be interpreted as applicable only to external radiation exposure. However, authoritative long-term monitoring data from QNPP indicate that overall radioactive releases, including those contribute to internal exposure, are far below the limits specified in national standards (GB 6249-2011) (29).

A key limitation is the weakened statistical power for radiosensitive cancers (leukemia and thyroid cancer) due to the small number of deaths. During 2012–2022, only 253 leukemia deaths (crude mortality rate: 6.05/100,000) and 14 thyroid cancer deaths (crude mortality rate: 0.33/100,000) were reported in Haiyan County. The small number of events yields unstable estimates, and thus reduce the ability of the current study to detect modest associations.

The crude and age-standardized cancer mortality rates in Haiyan County were slightly higher than those reported for Zhejiang's cancer registration areas (2010–2014). These provincial comparisons are descriptive rather than inferential, as the study did not adjust for confounding factors such as socioeconomic status, lifestyle patterns, and access to medical resources, which may vary between regions (32). Such regional differences are far more likely attributable to socioeconomic and lifestyle factors than to environmental radiation exposure.

### 4.5 Implications and future directions

This study provides critical baseline data for radiation protection and long-term monitoring around QNPP. Given the limitations identified, future research should address gaps to provide a more holistic evaluation of radiation risks, including (1) Expand exposure assessment to include internal pathways through targeted sampling of food, water, soil, and biological specimens; (2) Extend follow-up periods to address the long latency of radiation-induced cancers and improve statistical power for radiosensitive subtypes; (3) Incorporate individual-level data to consolidate variability in radiation sensitivity and confounding factors; (4) Integrate reproductive health outcomes as sensitive indicators of radiation effects, leveraging collaboration with local maternal and child health care institutions; (5) Conduct continuous long-term monitoring to track potential cumulative effects of extended nuclear power plant operation.

Despite these limitations, the core conclusion remains robust: Environmental radiation around QNPP Phase I remains at natural background levels before and after service extension, with *AED*_e_ and *ER*_e_ well below national and international limits. No statistically significant association was detected between radiation exposure and cancer mortality trends in surrounding residents. These findings do not provide evidence of adverse health or environmental impacts related to external radiation exposure around QNPP.

## 5 Conclusions

This study monitored ambient external radiation around QNPP Phase I from 2010 to 2023 (pre-extension: 2010–2021; post-extension: 2022–2023) and analyzed cancer mortality trends among Haiyan County residents (2012–2022). Ambient external radiation remained within natural background levels, with mean annual *CAD* of 0.350 mSv pre-extension and 0.675 mSv post-extension. Corresponding *AED*_e_ and *ER*_e_ were well below national and international regulatory limits.

Crude and age-standardized cancer mortality rates in the study area were slightly higher than Zhejiang Provincial averages—attributed to non-radiation confounders—with non-statistically significant downward trends with time for overall cancer, leukemia, and thyroid cancer. As an ecological descriptive study focusing solely on external radiation and cancer mortality, no significant association was found between QNPP operation (pre- and post-extension) and cancer mortality trends.

Key limitations of this study include the ecological design which precludes causal inference; the absence of assessment on internal radiation exposure; the restricted range of health outcomes analyzed (excluding reproductive health indicators); limited statistical power for radiosensitive cancers subtypes; and the relatively short post-extension follow-up period compared to typical cancer latency. Nevertheless, the long-term operational history of QNPP Phase I, together with our monitoring findings, indicates no detectable adverse environmental or public health impacts associated with external radiation exposure during the study period.

Continuous long-term monitoring is required to address potential delayed risks. Future research should expand exposure assessment, integrate broader health endpoints (notably reproductive outcomes), and incorporate individual-level data to strengthen radiation protection evidence.

These findings do not provide evidence of adverse environmental or public health impacts associated with external radiation exposure around QNPP Phase I within the study period. Absence of evidence is not evidence of absence, and continued long-term monitoring remains necessary.

## Data Availability

The original contributions presented in the study are included in the article/supplementary material, further inquiries can be directed to the corresponding author.
